# Epidural Catheter Placement in Morbidly Obese Parturients with the Use of an Epidural Depth Equation prior to Ultrasound Visualization

**DOI:** 10.1155/2013/695209

**Published:** 2013-07-25

**Authors:** Sukhdip Singh, Keith M. Wirth, Amy L. Phelps, Manasi H. Badve, Tanmay H. Shah, Neera Sah, Manuel C. Vallejo

**Affiliations:** ^1^Magee-Womens Hospital, Pittsburgh, PA 15213, USA; ^2^Department of Anesthesiology, University of Pittsburgh and Duquesne University School of Business, Pittsburgh, PA 15282, USA; ^3^St. Margaret's Hospital, Pittsburgh, PA 15203, USA; ^4^Department of Anesthesiology, Magee-Womens Hospital of UPMC, 300 Halket Street, Pittsburgh, PA 15213, USA

## Abstract

*Background*. Previously, Balki determined the Pearson correlation coefficient with the use of ultrasound (US) was 0.85 in morbidly obese parturients. We aimed to determine if the use of the epidural depth equation (EDE) in conjunction with US can provide better clinical correlation in estimating the distance from the skin to the epidural space in morbidly obese parturients. *Methods*. One hundred sixty morbidly obese (≥40 kg/m^2^) parturients requesting labor epidural analgesia were enrolled. Before epidural catheter placement, EDE was used to estimate depth to the epidural space. This estimation was used to help visualize the epidural space with the transverse and midline longitudinal US views and to measure depth to epidural space. The measured epidural depth was made available to the resident trainee before needle insertion. Actual needle depth (ND) to the epidural space was recorded. *Results*. Pearson's correlation coefficients comparing actual (ND) versus US estimated depth to the epidural space in the longitudinal median and transverse planes were 0.905 (95% CI: 0.873 to 0.929) and 0.899 (95% CI: 0.865 to 0.925), respectively. *Conclusion*. Use of the epidural depth equation (EDE) in conjunction with the longitudinal and transverse US views results in better clinical correlation than with the use of US alone.

## 1. Introduction 

The prevalence of obesity has grown to epidemic proportions over the past 20 years, with estimates of at least 1.6 billion overweight and 400 million obese adults worldwide [1]. Many parturients gain a significant amount of weight during pregnancy, and hence, many patients satisfy the requirement for obesity with a BMI > 30 kg/m^2^, making appropriate management an important concern for obstetric clinicians worldwide [2].

The physiological and anatomical changes associated with pregnancy, along with morbid obesity, introduce a number of unique considerations for anesthesia management. Compared to normal weight parturients, the obese parturient is prone to a number of complications during pregnancy and delivery including gestational hypertension, gestational diabetes, preeclampsia, shoulder dystocia, fetal macrosomia, and higher rates of Cesarean section along with increased operative time [3–5]. Difficult intubation in the morbidly obese parturient during induction of general anesthesia is one of the most recognized causes of anesthesia-related maternal mortality, with a reported 1 : 250 incidence of failed intubation in the obstetric population, compared to 1 : 2,280 incidence in the general population [6–8]. Increases in Mallampati scores have been correlated with gain in body weight, most likely due to the excess adipose tissue and edema of the upper airway commonly seen in the obese and pregnant population [9]. The obese parturient is at greater risk for pulmonary aspiration and inadequate ventilation [10]. Thus, placement of a well-functioning and reliable epidural catheter is paramount in preventing the increased risk in maternal morbidity and mortality inherent in the morbidly obese parturient. 

While regional anesthesia can offer these advantages over general anesthesia, the increased amount of subcutaneous and epidural fat in the obese population can pose a significant challenge to successful epidural catheter placement. Before US visualization, palpation of bony landmarks was the only available technique for identifying the lumbar interspinous spaces. Stiffler et al. [11] reported difficulty in palpating landmarks in 5% of patients with normal BMI, 33% in those who were overweight, and in 68% of obese patients. The spinous processes of some obese patients can be located more than 5 cm from the skin, with the ligamentum flavum as deep as 8 cm, and at extremes of 11-12 cm deep [12]. Grau et al. [13] determined that at term, the optimum puncture area on the skin for epidural cannulation is smaller, the soft tissue channel between the spinal processes is narrower, and the epidural space is also narrower. Each of these changes would most likely be exaggerated in those patients who are morbidly obese. Other reasons include difficulty in patient positioning and an increased likelihood for false positivity to the loss of resistance technique when locating the epidural space [13, 14]. 

The use of ultrasonography (US) for placing epidural catheters has become increasingly popular and has been shown to reduce epidural catheter failure rates and placement attempts [15, 16]. US can be used to identify the epidural space, localize midline, provide an estimation of depth from skin to the epidural space, and estimate the point of insertion and the angle of needle insertion [16–19]. Prepuncture visualization of the epidural space has been shown to decrease the number of attempts for epidural placement and decrease the incidence of accidental dural puncture, especially among new resident trainees [12, 15, 16]. The presence of increased adipose tissue makes US visualization of the epidural space more challenging, and a significant problem when using US in obese parturients is that decreased visualization of the epidural space makes estimation of the distance from the skin to the epidural space less predictable [12].

In a previous study, we investigated the use of US for epidural catheter placement in laboring parturients, found high correlation between estimated US depth and actual needle depth (ND); (Pearson's correlation coefficient ≥0.91) [16], and derived an epidural depth equation (EDE) using stepwise multivariate linear regression for predicting the distance from the skin to the epidural space in the lower lumbar intervertebral area.

In a study by Balki et al. [20] on morbidly obese parturients using US, they found a Pearson's correlation coefficient of 0.85 between the US estimated distance to the epidural space and the actual needle depth (ND). The aim of this study is to determine if EDE to estimate the depth of the epidural space before US visualization could improve imaging and result in better clinical correlation in morbidly obese parturients.

## 2. Methods

With local investigative review board approval and informed verbal and written consent, morbidly obese parturients (BMI ≥ 40 kg/m^2^) who requested labor epidural analgesia or scheduled for elective Cesarean section were recruited into the study. Exclusion criteria included patients with severe preeclampsia, a history of back surgery, significant scoliosis, BMI ≤ 40 kg/m^2^, and/or lumbar pathology.

### 2.1. Epidural Depth Equation Determination and Ultrasound Scanning

Prior to the US scanning and epidural catheter insertion, the estimated epidural depth was first calculated using the described epidural depth equation (EDE) derived from previous US study at our institution [16]:

(1) Epidural  Depth  (cm)=6.63−[0.07×Ht(in)]+[0.02×Wt(lbs)].

**Figure 1:**
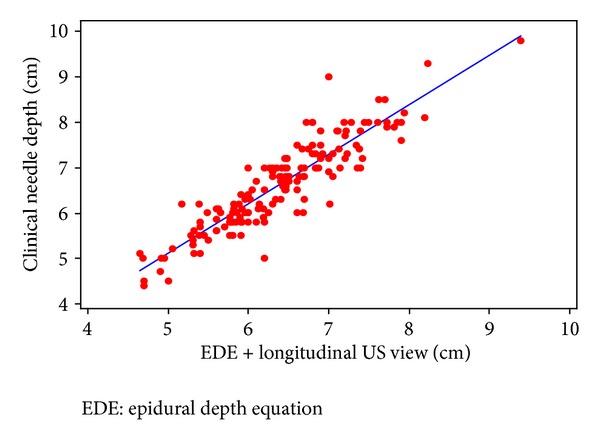
Epidural depth equation plus longitudinal US view versus clinical needle depth.

**Figure 2:**
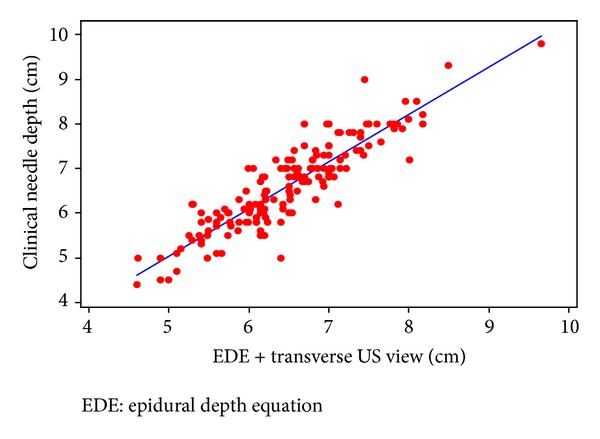
Epidural depth equation plus transverse US view versus clinical needle depth.

Then, the L3-4 or L4-5 intervertebral space was determined by palpation of the iliac crest, and on Tuffier's line, the vertebral interspace was identified and marked with an indelible marker. This mark was used to visualize both the longitudinal median and transverse US planes, confirm midline, and determine the final insertion point at the intervertebral space which gave the best view of the ligamentum flavum. With prior knowledge of the calculated epidural depth, the parturient had US visualization in the longitudinal median and transverse planes with estimation of the distance from skin to ligamentum flavum (posterior dura) before insertion of the epidural catheter (Figures 1 and 2) by the primary investigator (MV). The SonoSite S-Nerve US system (SonoSite, Bothell, WA, USA) with a 2–5 MHz curved array probe was used for US scanning. A resident trainee who was supervised by the primary investigator (MV) placed the epidural catheter with knowledge of the calculated epidural depth, US longitudinal median epidural depth, and US transverse epidural depth. Once the epidural catheter was placed in the epidural space, the actual clinical needle depth to the epidural space (ND) was recorded.

### 2.2. Epidural Technique

The study protocol followed the standard labor epidural technique at our institution, with the only exception being the use of EDE to initially estimate needle depth, along with US views (transverse and longitudinal) to measure the depth to the epidural space, determine midline, insertion point, and needle direction before epidural catheter insertion. The sitting position was maintained throughout US visualization and epidural catheter placement. 

Using a sterile technique, the epidural catheter was placed using the midline approach through a 17-gauge Tuohy needle at the L3-4 or L4-5 vertebral interspace using a loss of resistance to saline technique. The epidural catheter (Arrow FlexTip Plus, Arrow International, Reading, PA, USA) was inserted 5 cm into the epidural space and then secured with adhesive dressing and tape. All patients were given an initial bolus of 10 mL 0.0825% bupivacaine and fentanyl 100 *μ*g and placed on a patient controlled epidural analgesia (PCEA) infusion. PCEA parameters included continuous epidural infusion of 8 mL/hr, PCEA demand bolus dose of 8 mL, PCEA demand bolus dose lockout every 8 minutes, and PCEA 1-hour total lockout of 24 mL. Patients undergoing elective Cesarean section had a spinal needle placed through the epidural needle and were dosed with 12 mg of 0.75% hyperbaric bupivacaine with 0.2 mg preservative free morphine and 20 *μ*g fentanyl.

An epidural insertion attempt was defined as advancement of the needle in an effort to enter the epidural space; a needle requiring withdrawal for redirection or reinsertion was counted as an additional attempt. A failed epidural was defined as an epidural catheter requiring replacement during labor. Early and late failures were defined according to whether the catheter required replacement within or after the first 90 min following insertion, respectively. An attending staff anesthesiologist who was blinded to whether EDE + US views were obtained made the decision regarding epidural catheter replacement. The visual analogue scale (VAS) score (0 = no pain, 10 = worst pain) was used to assess pain before and after epidural activation and when assessing patients for inadequate analgesia. A failed epidural was defined as a block providing inadequate analgesia (VAS ≥ 4/10) despite the following sequential steps: (1) a 10 mL bolus of the epidural infusion mix and reassessment at 15 min; (2) a second 10 mL bolus of the epidural infusion mix and reassessment at 15 min; and (3) a 5 mL 1.5% lidocaine bolus.

Measured variables included demographic and obstetric data (age, height, and weight), number of pregnancies, parity, cervical dilation, failed epidural rate, epidural insertion attempts (redirections), epidural placement attempts (reinsertions), staff interventions (need for the attending anesthesiologist's assistance during the placement attempt), number of additional epidural top-ups (boluses) required, accidental dural puncture (ADP) rate, and maternal delivery outcome (vaginal delivery, cesarean, and elective cesarean). The calculated epidural depth from EDE, US longitudinal median epidural depth (ligamentum flavum/posterior dura), US transverse epidural depth (ligamentum flavum/posterior dura), and the actual clinical needle depth (ND) to the epidural space, measured using a sterile ruler to the nearest 0.1 cm, were also recorded.

### 2.3. Statistical Analysis

Balki et al. [20] found that the Pearson correlation coefficient between the UD and ND depth was 0.85; 95% CI, 0.75–0.91. We believe that the use of EDE + US would result in a correlation coefficient of approximately 0.91, as reported in our previous study [16]. The distribution of sample correlation coefficients was not normally dispersed, and confidence intervals for correlation coefficients were not symmetric. Therefore, in order to keep the lower bound estimate within 0.04 of a correlation of 0.91, and to maintain a 95% confidence level, 140 patients would need to be sampled. To allow for patients who may not complete the study, 160 patients were enrolled. 

Demographic data was analyzed using descriptive statistics, including mean (±SD) for interval data, percentages for nominal data, and median (range) for ordinal data. Pearson's correlation coefficient was calculated for epidural distance measurements which included actual clinical epidural needle depth (ND) and the epidural depth equation (EDE), ND and prior EDE + US midline longitudinal plane view, and ND and prior EDE + US transverse plane view.

## 3. Results

From August 2010 to June 2011, a total of 160 parturients were studied. All women who were approached participated in and completed the study. Maternal demographic data is presented in Table 1. There were 9 epidural block failures: two were early failures (<90 minutes), and 7 were late failures (>90 minutes). No patient had more than one failed epidural block. There was only one recognized accidental dural puncture (ADP) which went on to become a postdural puncture headache (PDPH) and required a therapeutic epidural blood patch (Table 2). The epidural needle placement was done without reinsertions in 92% of the patients, with no need to redirect the needle in 54% of the parturients. The maximum number of reinsertions at the same intervertebral level was four, and 90% of the catheters were successfully placed in three or fewer redirection attempts through the same puncture site.

Mean depths to the ligamentum flavum/dorsal dura as measured by longitudinal, median, and transverse US planes, and the depth estimated by EDE are presented in Table 2. Both the longitudinal median and transverse US planes had high correlation with actual clinical depth (ND) to the epidural space. Pearson's correlation coefficients comparing clinical depth to longitudinal median and transverse US plane views were 0.905; 95% CI, 0.873 to 0.929 and 0.899; 95% CI, 0.865 to 0.925, respectively. Pearson's correlation coefficient comparing the transverse US plane to the longitudinal median US plane was 0.948; 95% CI, 0.930 to 0.961. Graphical representations of EDE + US views versus ND, with best-fit lines, in the longitudinal and transverse planes are shown in Figures 1 and 2. 

## 4. Discussion

Among the general population, the incidence of accidental dural puncture (ADP) without the use of US has been reported as ranging from 1% to 5%. Balki et al. [20] had no dural punctures in a series of 46 obese patients when they used US to estimate epidural depth, while in our study the incidence was 0.6%. The preexisting epidural catheter failure rate has been estimated as 1.5–7%, and the rate of conversion to general anesthesia in patients requiring cesarean sections ranges from 4.3% to 6.0% compared to 4.4% in our study [12, 15, 21, 22]. The incidence of difficult epidural catheter placement and early failure is significantly more likely among the morbidly obese population. Morbidly obese parturients have a higher incidence of initial epidural failure rate, with reports as high as 42%, versus 6% in the general population [23]. In a comparison of morbidly obese to a control population, Vricella et al. [24] found a higher incidence of complicated placement (5.6% versus 0%), failure to establish (2.0% versus 0%), and insufficient duration (4.0% versus 0%) of regional anesthesia. With a complicated placement of regional anesthesia being defined as >3 attempts, our incidence of complicated placements was 3.8%, compared to the 5.6% in the study by Vricella et al. [24]. Our study showed a 1.6% incidence of failed initial attempt and no patients with failure to establish an epidural block which is again similar to the results of Balki et al. [20] who also had effective pain relief in all patients.

The correlation between estimated epidural depth using US and clinical epidural depth has been reported to range between 0.881 and 0.96 in the general obstetric population, with the mean clinical epidural depth being less than the US estimate [18, 25, 26]. In Balki et al.'s [20] study, the correlation between UD and ND in an obese population was reported to be 0.85, with a 95% confidence interval of 0.75–0.91. While this showed that epidural depth estimation with US is clinically useful in the obese parturient population, the correlation between UD and ND is still less than that of the general nonobese population. The visibility of the ligamentum flavum via ultrasonography decreases as women progress through pregnancy and has been attributed to weight gain and edema [13]. We believe the lower correlation between UD and ND found by Balki et al. [20] in the obese population is, in part, due to similar visualization difficulties associated with the increased adipose tissue of morbidly obese parturients. We sought to increase this correlation, and therefore its clinical applicability, by introducing the use of EDE prior to US scanning. EDE was derived in our previous study, using a using stepwise multivariate linear regression, with height and weight (= BMI) as the variables used for prediction [16]. BMI has been shown to be the most reliable indicator of the skin to lumbar epidural space distance [27–29].

Using EDE prior to US scanning, we found that the Pearson correlation coefficients between EDE + US and ND were 0.905 and 0.899 in the longitudinal and transverse planes, respectively. Each of these correlations was better than the 0.85 correlation found in the study by Balki et al. [20], suggesting that EDE can be a useful tool for assessing epidural catheter depth in the morbidly obese parturient. We found that the greatest correlation between the US estimation and the clinical needle depth (ND) was in the longitudinal US plane, while previous studies have suggested that the transverse plane provides the most reliable information [18, 20]. Nonetheless, both the transverse and longitudinal US views should be used as both views can confirm midline, estimate needle depth, and determine the angle of needle insertion [16]. 

Previous studies have reported the mean depth to the epidural space as ranging from 4.8 to 5.6 cm in obstetric populations with variable BMI [29–31]. In Balki et al.'s [20] study, the mean depth to the epidural space in an obese obstetric population was reported as 6.6 cm (range 4.5–8.5 cm), which is the same mean calculated in our study, 6.6 cm (range 4.4–9.8 cm). The depth to the epidural space was more than 8 cm in 4.3% of the patients in our study, less than the 14% reported by Balki et al. [20]. Nine parturients received multiple epidural catheter placements due to inadequate relief (no more than 2 placements), but 7 of these were defined as “late” failures, which are more likely due to dislodgement of the catheter rather than improper placement. This suggests that the use of EDE prior to prepuncture US can provide more accurate prediction of depth to the epidural space.

## 5. Conclusion

The use of the epidural depth equation (EDE) prior to ultrasound visualization in the longitudinal and transverse US views results in better clinical correlation than with the use of ultrasound alone.

## Figures and Tables

**Table 1:** Maternal demographic data.

Age (years)	28.9 ± 6.4
Height (cm)	164.8 ± 7.0
Weight (kg)	122.6 ± 17.8
BMI (kg/m^2^)	45.0 ± 5.0
Gestation (weeks)	38.8 ± 2.2
Gravidy	2 (1–11)
Parity	0 (0–9)

Data are mean ± SD or median with range in parenthesis.

**Table 2:** Maternal outcome data.

Epidural depth (cm)	
Clinical	6.6 ± 1.0
Equation	6.5 ± 0.7
US longitudinal	6.4 ± 0.8
US transverse	6.5 ± 0.8
Failed epidural	9 (5.6%)
Failed early epidural	2 (1.3%)
No. of epidural attempts	1 (1–4)
Without redirection	86 (54%)
No. of epidural placements	1 (1-2) IQ [1-2]
Without reinsertion	147 (92%)
Staff intervention	32 (20%)
Additional top-ups (boluses)	0 (0–8)
Accidental dural puncture	3 (1.9%)
Delivery type	
Vaginal	108 (67.5%)
Cesarean	41 (25.6%)
Elective Cesarean	11 (6.9%)
Postdural puncture headache	1 (0.6%)
Epidural blood patch	1 (0.6%)

Data are mean ± SD, or median with range in parenthesis, or number with percentage in parenthesis. IQ: interquartile with 25th and 75th quartiles in brackets.
